# Who Is the Best Player Ever? A Complex Network Analysis of the History of Professional Tennis

**DOI:** 10.1371/journal.pone.0017249

**Published:** 2011-02-09

**Authors:** Filippo Radicchi

**Affiliations:** Department of Chemical and Biological Engineering, Northwestern University, Evanston, Illinois, United States of America; University of Maribor, Slovenia

## Abstract

We considered all matches played by professional tennis players between 1968 and2010, and, on the basis of this data set, constructed a directed and weighted network of contacts. The resulting graph showed complex features, typical of many real networked systems studied in literature. We developed a diffusion algorithm and applied it to the tennis contact network in order to rank professional players. *Jimmy Connors* was identified as the best player in the history of tennis according to our ranking procedure. We performed a complete analysis by determining the best players on specific playing surfaces as well as the best ones in each of the years covered by the data set. The results of our technique were compared to those of two other well established methods. In general, we observed that our ranking method performed better: it had a higher predictive power and did not require the arbitrary introduction of external criteria for the correct assessment of the quality of players. The present work provides novel evidence of the utility of tools and methods of network theory in real applications.

## Introduction

Social systems generally display complex features [1]. Complexity is present at the individual level: the behavior of humans often obeys complex dynamical patterns as for example demonstrated by the rules governing electronic correspondence [2]–[5]. At the same time, complexity is present also at the global level. This can be seen for example when social systems are mathematically represented in terms of graphs or networks, where vertices identify individuals and edges stand for interactions between pairs of social agents. Social networks are in most of the cases *scale-free* [6], indicating therefore a strong degree of complexity from the topological and global points of view.

During last years, the analysis of social systems has become an important topic of interdisciplinary research and as such has started to be not longer of interest to social scientists only. The presence of a huge amount of digital data, describing the activity of humans and the way in which they interact, has made possible the analysis of large-scale systems. This new trend of research does not focus on the behavior of single agents, but mainly on the analysis of the macroscopic and statistical properties of the whole population, with the aim to discover regularities and universal rules. In this sense, professional sports also represent optimal sources of data. Soccer [7]–[9], football [10], [11], baseball [12]–[15] and basketball [16], [17] are some remarkable cases in which network analysis revealed features not visible with traditional approaches. These are practical examples of the general outcome produced by the intense research activity of last years: network tools and theories do not serve only for descriptive purposes, but have also wide practical applicability. Representing a real system as a network allows in fact to have a global view of the system and simultaneously use the entire information encoded by its complete list of interactions. Particularly relevant results are those regarding: the robustness of networks under intentional attacks [18]; the spreading of viruses in graphs [19]; synchronization processes [20], social models [1], and evolutionary and coevolutionary games [21], [22] taking place on networks. In this context fall also ranking techniques like the PageRank algorithm [23], where vertices are ranked on the basis of their “centrality” in a diffusion process occurring on the graph. Diffusion algorithms, originally proposed for ranking web pages, have been recently applied to citation networks [24]. The evaluation of the popularity of papers [25], journals [26], [27] and scientists [28] is performed not by looking at local properties of the network (i.e., number of citations) but by measuring their degree of centrality in the flow of information diffusing over the entire graph. The use of the whole network leads to better evaluation criteria without the addition of external ingredients because the complexity of the citation process is encoded by the topology of the graph.

In this paper we continue in this direction of research and present a novel example of a real system, taken from the world of professional sports, suitable for network representation. We consider the list of all tennis matches played by professional players during the last 43 years (1968–2010). Matches are considered as basic contacts between the actors in the network and weighted connections are drawn on the basis of the number of matches between the same two opponents. We first provide evidence of the complexity of the network of contacts between tennis players. We then develop a ranking algorithm similar to PageRank and quantify the importance of tennis players with the so-called “prestige score”. The results presented here indicate once more that ranking techniques based on networks outperform traditional methods. The prestige score is in fact more accurate and has higher predictive power than well established ranking schemes adopted in professional tennis. More importantly, our ranking method does not require the introduction of external criteria for the assessment of the quality of players and tournaments. Their importance is self-determined by the various competitive processes described by the intricate network of contacts. Our algorithm does nothing more than taking into account this information.

## Methods

### Data set

**Figure 1:**
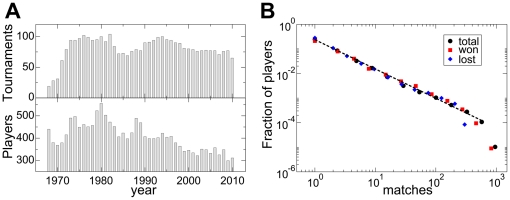
Properties of the data set. In panel a, we report the total number of tournaments (top panel) and players (bottom panel) as a function of time. In panel b, we plot the fraction of players having played (black circles), won (red squares) and lost (blue diamonds) a certain number of matches. The black dashed line corresponds to the best power-law fit with exponent consistent with the value 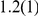.

Data were collected from the web site of the Association of Tennis Professionals (ATP, www.atpworldtour.com). We automatically downloaded all matches played by professional tennis players from January 1968 to October 2010. We restrict our analysis only to matches played in Grand Slams and ATP World Tour tournaments for a total of 3640 tournaments and 133261 matches. For illustrative purposes, in the top plot of the panel a of Figure 1, we report the number of tournaments played in each of the years covered by our data set. With the exception of the period between 1968 and 1970, when ATP was still in its infancy, about 75 tournaments were played each year. Two periods of larger popularity were registered around years 1980 and 1992 when more than 90 tournaments per year were played. The total number of different players present in our data set is 3700, and in the bottom plot of panel a of Figure 1 we show how many players played at least one match in each of the years covered by our analysis. In this case, the function is less regular. On average, 400 different players played in each of the years between 1968 and 1996. Large fluctuations are anyway visible and a very high peak in 1980, when more than 500 players participated in ATP tournaments, is also present. Between 1996 and 2000, the number of players decreased from 400 to 300 in an almost linear fashion. After that, the number of participants in ATP tournaments started to be more constant with small fluctuations around an average of about 300 players.

### Network representation

**Figure 2:**
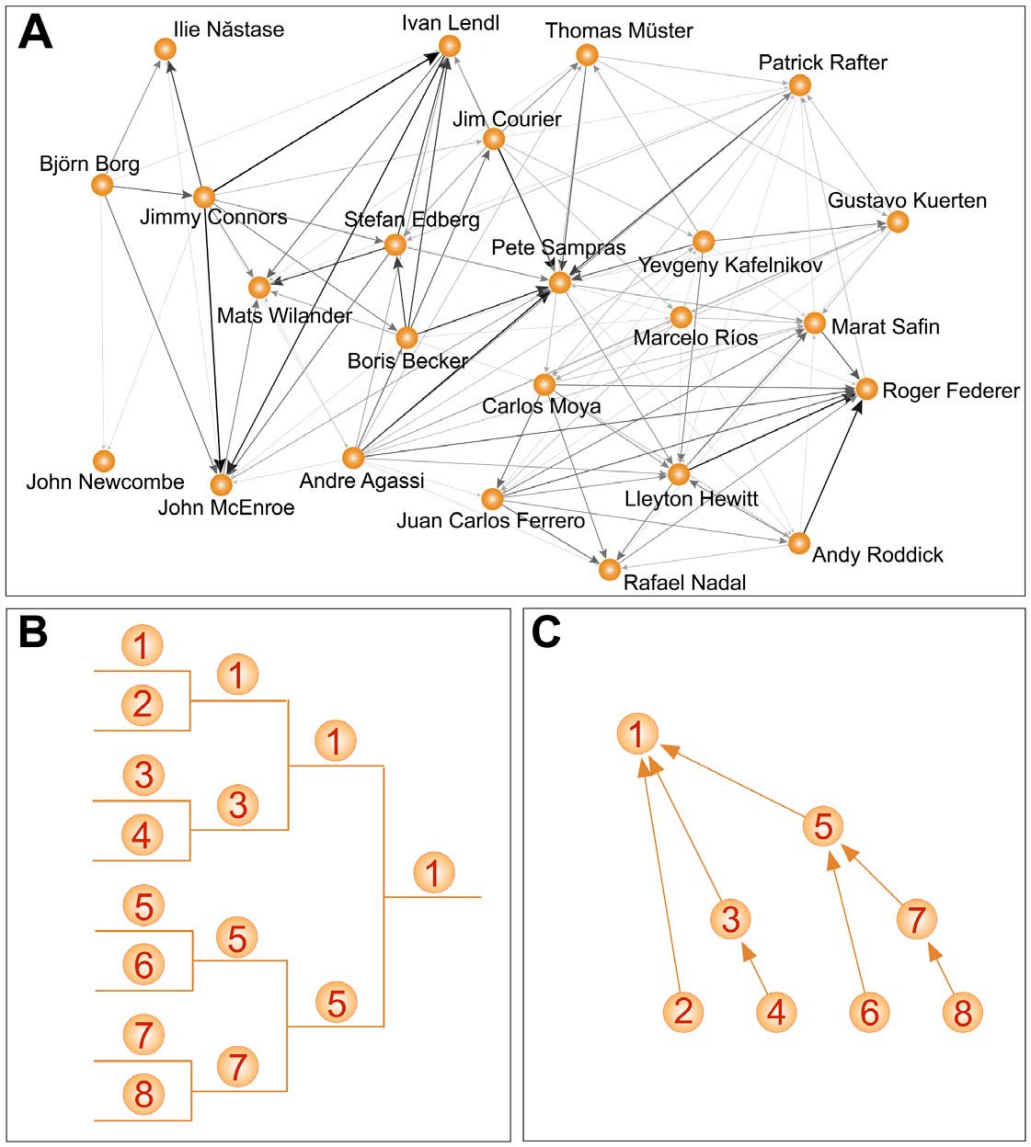
Top player network and scheme for a single tournament. In panel a, we draw the subgraph of the contact network restricted only to those players who have been number one in the ATP ranking. Intensities and widths are proportional to the logarithm of the weight carried by each directed edge. In panel b, we report a schematic view of the matches played during a single tournament, while in panel c we draw the network derived from it.

We represent the data set as a network of contacts between tennis players. This is a very natural representation of the system since a single match can be viewed as an elementary contact between two opponents. Each time the player 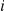 plays and wins against player 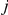, we draw a directed connection from 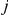 to 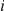 [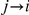, see Figure 2]. We adopt a weighted representation of the contacts [29], by assigning to the generic directed edge 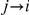 a weight 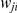 equal to the number of times that player 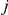 looses against player 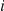. Our data are flexible and allow various levels of representation by including for example only matches played in a certain period of time, on a certain type of surface, etc. An example is reported in panel a of Figure 2 where the network of contacts is restricted only to the 24 players having been number one in the official ATP ranking. In general, networks obtained from the aggregation of a sufficiently high number of matches have topological complex features consistent with the majority of networked social systems so far studied in literature [30], [31]. Typical measures revealing complex structure are represented by the probability density functions of the in- and out-strengths of vertices [29], both following a clear power-law behavior [see Figure 1, panel b]. In our social system, this means that most of the players perform a small number of matches (won or lost) and then quit playing in major tournaments. On the other hand, a small set of top players performs many matches against worse opponents (generally beating them) and also many matches (won or lost) against other top players. This picture is consistent with the so-called “Matthew effect” in career longevity recently observed also in other professional sports [12], [15].

### Prestige score

The network representation can be used for ranking players. In our interpretation, each player in the network carries a unit of “tennis prestige” and we imagine that prestige flows in the graph along its weighted connections. The process can be mathematically solved by determining the solution of the system of equations
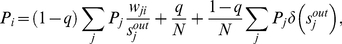
(1)valid for all nodes 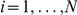, with the additional constraint that 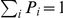. 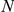 indicates the total number of players (vertices) in the network, while 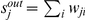 is the out-strength of the node 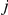 (i.e., the sum of the weight of all edges departing from vertex 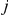). 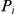 is the “prestige score” assigned to player 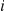 and represents the fraction of the overall tennis prestige sitting, in the steady state of the diffusion process, on vertex 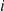. In Eqs. (1), 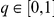 is a control parameter which accounts for the importance of the various terms contributing to the score of the nodes. The term 
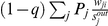
 represents the portion of score received by node 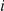 in the diffusion process: vertices redistribute their entire credit to neighboring nodes proportionally to the weight of the connections linking to them. 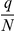 stands for a uniform redistribution of tennis prestige among all nodes according to which each player in the graph receives a constant and equal amount of credit. Finally the term 
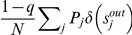
 [with 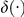 equal to one only if its argument is equal to zero, and zero otherwise] serves as a correction in the case of existence of dandling nodes (i.e., nodes with null out-strength), which otherwise would behave as sinks in the diffusion process. Our prestige score is analogous to the PageRank score [23], originally formulated for ranking web pages and more recently applied in different contexts.

In general topologies, analytical solutions of Eqs. (1) are hard to find. The stationary values of the scores 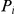
s can be anyway computed recursively, by setting at the beginning 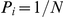 (but the results do not depend on the choice of the initial value) and iterating Eqs. (1) until they converge to values stable within *a priori* fixed precision.

### Single tournament

In the simplest case in which the graph is obtained by aggregating matches of a single tournament only, we can analytically determine the solutions of Eqs. (1). In a single tournament, matches are hierarchically organized in a binary rooted tree and the topology of the resulting contact network is very simple [see Figure 2, panels b and c]. Indicate with 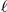 the number of matches that the winner of the tournament should play (and win). The total number of players present at the beginning of the tournament is 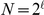. The prestige score is simply a function of 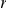, the number of matches won by a player, and can be denoted by 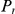. We can rewrite Eqs. (1) as
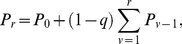
(2)where 
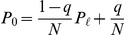
 and 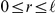. The score 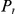 is given by the sum of two terms: 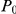 stands for the equal contribution shared by all players independently of the number of victories; 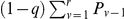 represents the score accrued for the number of matches won. The former system of equations has a recursive solution given by
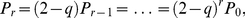
(3)which is still dependent on a constant that can be determined by implementing the normalization condition
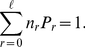
(4)


In Eq. (4), 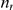 indicates the number of players who have won 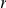 matches. We have 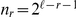 for 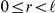 and 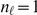 and Eqs. (3) and (4) allow to compute
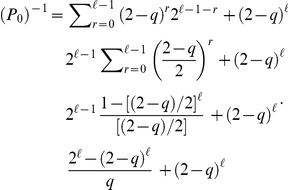



In the former calculations, we have used the well known identity 
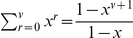
, valid for any 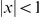 and 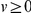, which respectively means 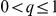 and 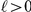 in our case. Finally, we obtain
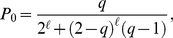
(5)which together with Eqs. (3) provides the solution
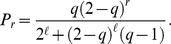
(6)


It is worth to notice that for 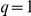, Eqs. (6) correctly give 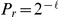 for any 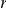, meaning that, in absence of diffusion, prestige is homogeneously distributed among all nodes. Conversely, for 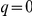 the solution is
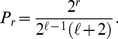
(7)


**Figure 3:**
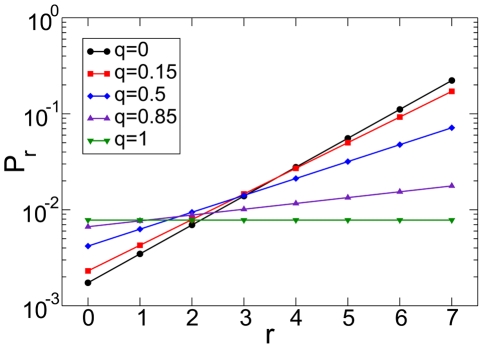
Prestige score in a single tournament. Prestige score 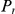 as a function of the number of victories 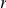 in a tournament with 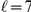 rounds (Grand Slam). Black circles are obtained from Eqs. (7) and valid for 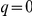. All other values of 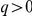 have been calculated from Eqs. (6): red squares stand for 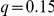, blue diamonds for 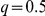, violet up-triangles for 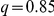 and green down-triangles for 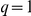.

In Figure 3, we plot Eqs. (6) and (7) for various values of 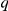. In general, sufficiently low values of 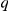 allow to assign to the winner of the tournament a score which is about two order of magnitude larger than the one given to players loosing at the first round. The score of the winner is an exponential function of 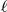, the length of the tournament. Grand Slams have for instance length 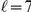 and their relative importance is therefore two or four times larger than the one of other ATP tournaments, typically having lengths 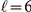 or 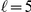.

## Results

We set 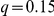 and run the ranking procedure on several networks derived from our data set. The choice 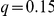 is mainly due to tradition. This is the value originally used in the PageRank algorithm [23] and then adopted in the majority of papers about this type of ranking procedures [25]–[28]. It should be stressed that 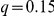 is also a reasonable value because it ensures a high relative score for the winner of the tournament as stated in Eqs. (6).

**Figure 4:**
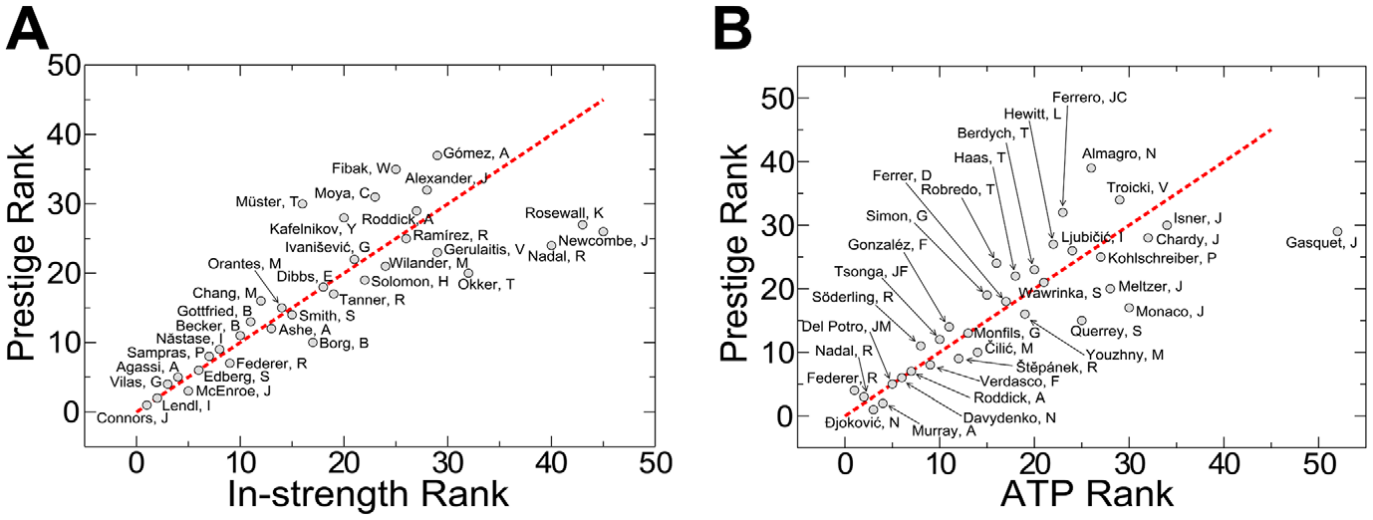
Relation between prestige rank and other ranking techniques. In panel a, we present a scatter plot of the prestige rank *versus* the rank based on the number of victories (i.e., in-strength). Only players ranked in the top 30 positions in one of the two lists are reported. Rank positions are calculated on the network corresponding to all matches played between 1968 and 2010. In panel b, a similar scatter plot is presented, but now only matches of year 2009 are considered for the construction of the network. Prestige rank positions are compared with those assigned by ATP.

In Table 1, we report the results obtained from the analysis of the contact network constructed over the whole data set. The method is very effective in finding the best players of the history of tennis. In our top 10 list, there are 9 players having been number one in the ATP ranking. Our ranking technique identifies *Jimmy Connors* as the best player of the history of tennis. This could be *a posteriori* justified by the extremely long and successful career of this player. Among all top players in the history of tennis, *Jimmy Connors* has been undoubtedly the one with the longest and most regular trend, being in the top 10 of the ATP year-end ranking for 16 consecutive years (1973–998). Prestige score is strongly correlated with the number of victories, but important differences are evident when the two techniques are compared. Panel a of Figure 4 shows a scatter plot, where the rank calculated according to our score is compared to the one based on the number of victories. An important outlier is this plot is represented by the *Rafael Nadal*, the actual number one of the ATP ranking. *Rafael Nadal* occupies the rank position number 40 according to the number of victories obtained in his still young career, but he is placed at position number 24 according to prestige score, consistently with his high relevance in the recent history of tennis. A similar effect is also visible for *Björn Borg*, whose career length was shorter than average. He is ranked at position 17 according to the number of victories. Prestige score differently is able to determine the undoubted importance of this player and, in our ranking, he is placed among the best 10 players of the whole history of professional tennis.

**Table 1:** Top 30 players in the history of tennis.

Rank	Player	Country	Hand	Start	End
1	Jimmy Connors	United States	L	1970	1996
2	Ivan Lendl	United States	R	1978	1994
3	John McEnroe	United States	L	1976	1994
4	Guillermo Vilas	Argentina	L	1969	1992
5	Andre Agassi	United States	R	1986	2006
6	Stefan Edberg	Sweden	R	1982	1996
7	Roger Federer	Switzerland	R	1998	2010
8	Pete Sampras	United States	R	1988	2002
9	Ilie N 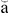 stase	Romania	R	1968	1985
10	Björn Borg	Sweden	R	1971	1993
11	Boris Becker	Germany	R	1983	1999
12	Arthur Ashe	United States	R	1968	1979
13	Brian Gottfried	United States	R	1970	1984
14	Stan Smith	United States	R	1968	1985
15	Manuel Orantes	Spain	L	1968	1984
16	Michael Chang	United States	R	1987	2003
17	Roscoe Tanner	United States	L	1969	1985
18	Eddie Dibbs	United States	R	1971	1984
19	Harold Solomon	United States	R	1971	1991
20	Tom Okker	Netherlands	R	1968	1981
21	Mats Wilander	Sweden	R	1980	1996
22	Goran Ivaniševi 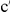	Croatia	L	1988	2004
23	Vitas Gerulaitis	United States	R	1971	1986
24	Rafael Nadal	Spain	L	2002	2010
25	Raúl Ramirez	Mexico	R	1970	1983
26	John Newcombe	Australia	R	1968	1981
27	Ken Rosewall	Australia	R	1968	1980
28	Yevgeny Kafelnikov	Russian Federation	R	1992	2003
29	Andy Roddick	United States	R	2000	2010
30	Thomas Müster	Austria	L	1984	1999

Players having been at the top of ATP ranking are highlighted in gray. From left to right we indicate for each player: rank position according to prestige score, full name, country of origin, the hand used to play, and the years of the first and last ATP tournament played.

In general, players still in activity are penalized with respect to those who have ended their careers. Prestige score is in fact strongly correlated with the number of victories [see panel a of Figure 4] and still active players did not yet played all matches of their career. This bias, introduced by the incompleteness of the data set, can be suppressed by considering, for example, only matches played in the same year. Table 2 shows the list of the best players of the year according to prestige score. It is interesting to see how our score is effective also here. We identify *Rod Laver* as the best tennis player between 1968 and 1971, period in which no ATP ranking was still established. Similar long periods of dominance are also those of *Ivan Lendl* (1981–1986), *Pete Sampras* (1992–1995) and *Roger Federer* (2003–2006). For comparison, we report the best players of the year according to ATP (year-end rank) and ITF (International Tennis Federation, www.itftennis.com) rankings. In many cases, the best players of the year are the same in all lists. Prestige rank seems however to have a higher predictive power by anticipating the best player of the subsequent year according to the two other rankings. *John McEnroe* is the top player in our ranking in 1980 and occupies the same position in the ATP and ITF lists one year later. The same happens also for *Ivan Lendl*, *Pete Sampras*, *Roger Federer* and *Rafael Nadal*, respectively best players of the years 1984, 1992, 2003 and 2007 according to prestige score, but only one year later placed at the top position of ATP and ITF rankings. The official ATP rank and the one determined on the basis of the prestige score are strongly correlated, but small differences between them are very interesting. An example is reported in panel b of Figure 4, where the prestige rank calculated over the contact network of 2009 is compared with the ATP rank of the end of the same year (official ATP year-end rank as of December 28, 2009). The top 4 positions according to prestige score do not corresponds to those of the ATP ranking. The best player of the year, for example, is *Novak Djokovi* 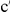 instead of *Roger Federer*.

**Table 2:** Best players of the year.

Year	Prestige	ATP year-end	ITF
1968	Rod Laver	-	-
1969	Rod Laver	-	-
1970	Rod Laver	-	-
1971	Ken Rosewall	-	-
1972	Ilie N 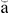 stase	-	-
1973	Tom Okker	Ilie N 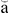 stase	-
1974	Björn Borg	Jimmy Connors	-
1975	Arthur Ashe	Jimmy Connors	-
1976	Jimmy Connors	Jimmy Connors	-
1977	Guillermo Vilas	Jimmy Connors	-
1978	Björn Borg	Jimmy Connors	Björn Borg
1979	Björn Borg	Björn Borg	Björn Borg
1980	John McEnroe	Björn Borg	Björn Borg
1981	Ivan Lendl	John McEnroe	John McEnroe
1982	Ivan Lendl	John McEnroe	Jimmy Connors
1983	Ivan Lendl	John McEnroe	John McEnroe
1984	Ivan Lendl	John McEnroe	John McEnroe
1985	Ivan Lendl	Ivan Lendl	Ivan Lendl
1986	Ivan Lendl	Ivan Lendl	Ivan Lendl
1987	Stefan Edberg	Ivan Lendl	Ivan Lendl
1988	Mats Wilander	Mats Wilander	Mats Wilander
1989	Ivan Lendl	Ivan Lendl	Boris Becker
1990	Stefan Edberg	Stefan Edberg	Ivan Lendl
1991	Stefan Edberg	Stefan Edberg	Stefan Edberg
1992	Pete Sampras	Jim Courier	Jim Courier
1993	Pete Sampras	Pete Sampras	Pete Sampras
1994	Pete Sampras	Pete Sampras	Pete Sampras
1995	Pete Sampras	Pete Sampras	Pete Sampras
1996	Goran Ivaniševi 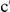	Pete Sampras	Pete Sampras
1997	Patrick Rafter	Pete Sampras	Pete Sampras
1998	Marcelo Ríos	Pete Sampras	Pete Sampras
1999	Andre Agassi	Andre Agassi	Andre Agassi
2000	Marat Safin	Gustavo Kuerten	Gustavo Kuerten
2001	Lleyton Hewitt	Lleyton Hewitt	Lleyton Hewitt
2002	Lleyton Hewitt	Lleyton Hewitt	Lleyton Hewitt
2003	Roger Federer	Andy Roddick	Andy Roddick
2004	Roger Federer	Roger Federer	Roger Federer
2005	Roger Federer	Roger Federer	Roger Federer
2006	Roger Federer	Roger Federer	Roger Federer
2007	Rafael Nadal	Roger Federer	Roger Federer
2008	Rafael Nadal	Rafael Nadal	Rafael Nadal
2009	Novak Djokovi 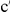	Roger Federer	Roger Federer
2010	Rafael Nadal	Rafael Nadal	Rafael Nadal

For each year we report the best player according to our ranking scheme and those of ATP and ITF. Best year-end ATP players are listed for all years from 1973 on. ITF world champions have started to be nominated since 1978 only.

We perform also a different kind of analysis by constructing networks of contacts for decades and for specific types of playing surfaces. According to our score, the best players per decade are (Tables S1, S2, S3, S4 list the top 30 players in each decade) : *Jimmy Connors* (1971–1980), *Ivan Lendl* (1981–1990), *Pete Sampras* (1991–2000) and *Roger Federer* (2001–2010). Prestige score identifies *Guillermo Vilas* as the best player ever in clay tournaments, while on grass and hard surfaces the best players ever are *Jimmy Connors* and *Andre Agassi*, respectively (see Tables S5, S6, S7 for the list of the top 30 players of a particular playing surface).

## Discussion

Tools and techniques of complex networks have wide applicability since many real systems can be naturally described as graphs. For instance, rankings based on diffusion are very effective since the whole information encoded by the network topology can be used in place of simple local properties or pre-determined and arbitrary criteria. Diffusion algorithms, like the one for calculating the PageRank score [23], were first developed for ranking web pages and more recently have been applied to citation networks [25]–[28]. In citation networks, diffusion algorithms generally outperform simple ranking techniques based on local network properties (i.e., number of citations). When the popularity of papers is in fact measured in terms of mere citation counts, there is no distinction between the quality of the citations received. In contrast, when a diffusion algorithm is used for the assessment of the quality of scientific publications, then it is not only important that popular papers receive many citations, but also that they are cited by other popular articles. In the case of citation networks however, possible biases are introduced in the absence of a proper classification of papers in scientific disciplines [32]. The average number of publications and citations strongly depend on the popularity of a particular topic of research and this fact influences the outcome of a diffusion ranking algorithm. Another important issue in paper citation networks is related to their intrinsic temporal nature: connections go only backward in time, because papers can cite only older articles and not *vice versa*. The anisotropy of the underlying network automatically biases any method based on diffusion. Possible corrections can be implemented: for example, the weight of citations may be represented by an exponential decaying function of the age difference between citing and cited papers [25]. Though these corrections can be reasonable, they are *ad hoc* recipes and as such may be considered arbitrary.

Here we have reported another emblematic example of a real social system suitable for network representation: the graph of contacts (i.e., matches) between professional tennis players. This network shows complex topological features and as such the understanding of the whole system cannot be achieved by decomposing the graph and studying each component in isolation. In particular, the correct assessment of players' performances needs the simultaneously consideration of the whole network of interactions. We have therefore introduced a new score, called “prestige score”, based on a diffusion process occurring on the entire network of contacts between tennis players. According to our ranking technique, the relevance of players is not related to the number of victories only but mostly to the quality of these victories. In this sense, it could be more important to beat a great player than to win many matches against less relevant opponents. The results of the analysis have revealed that our technique is effective in finding the best players of the history of tennis. The biases mentioned in the case of citation networks are not present in the tennis contact graph. Players do not need to be classified since everybody has the opportunity to participate to every tournament. Additionally, there is not temporal dependence because matches are played between opponents still in activity and the flow does not necessarily go from young players towards older ones. In general, players still in activity are penalized with respect to those who already ended their career only for incompleteness of information (i.e., they did not play all matches of their career) and not because of an intrinsic bias of the system. Our ranking technique is furthermore effective because it does not require any external criteria of judgment. As term of comparison, the actual ATP ranking is based on the amount of points collected by players during the season. Each tournament has an *a priori* fixed value and points are distributed accordingly to the round reached in the tournament. In our approach differently, the importance of a tournament is self-determined: its quality is established by the level of the players who are taking part of it.

In conclusion, we would like to stress that the aim of our method is not to replace other ranking techniques, optimized and almost perfected in the course of many years. Prestige rank represents only a novel method with a different spirit and may be used to corroborate the accuracy of other well established ranking techniques.

## Supporting Information

Table S1 **Top 30 players of the period 1971–1980.**
(PDF)

Table S2 **Top 30 players of the period 1981–190.**
(PDF)

Table S3 **Top 30 players of the period 1991–2000.**
(PDF)

Table S4 **Top 30 players of the period 2001–2010.**
(PDF)

Table S5 **Top 30 players of the history of tennis in tournaments played on clay.**
(PDF)

Table S6 **Top 30 players of the history of tennis in tournaments played on grass.**
(PDF)

Table S7 **Top 30 players of the history of tennis in tournaments played on hard surfaces.**
(PDF)

## References

[1] Castellano C, Fortunato S, Loreto V (2009). Statistical physics of social dynamics.. Rev Mod Phys.

[2] Barabási AL (2005). The origin of bursts and heavy tails in human dynamics.. Nature.

[3] Malmgren RD, Stouffer DB, Motter AE, Amaral LAN (2008). A poissonian explanation for heavy tails in e-mail communication.. Proc Natl Acad Sci USA.

[4] Radicchi F (2009). Human activity in the web.. Phys Rev E.

[5] Wu Y, Zhou C, Xiao J, Kurths J, Schellnhuber HJ (2010). Evidence for a bimodal distribution in human communication.. Proc Natl Acad Sci USA.

[6] Barabási AL, Albert R (1999). Emergence of scaling in random networks.. Science.

[7] Onody RN, de Castro PA (2004). Complex network study of brazilian soccer players.. Phys Rev E.

[8] Duch J, Waitzman JS, Amaral LAN (2010). Quantifying the performance of individual players in a team activity.. PLoS ONE.

[9] Heuer A, Müller C, Rubner O (2010). Soccer: Is scoring goals a predictable poissonian process?. EPL.

[10] Girvan M, Newman MEJ (2002). Community structure in social and biological networks.. Proc Natl Acad Sci USA.

[11] Ben-Naim E, Vazquez F, Redner S (2007). What is the most competitive sport?. J Korean Phys Soc.

[12] Petersen AM, Jung WS, Stanley HE (2008). On the distribution of career longevity and the evolution of home-run prowess in professional baseball.. EPL.

[13] Sire C, Redner S (2009). Understanding baseball team standings and streaks.. Eur Phys J B.

[14] Saavedra S, Powers S, McCotter T, Porter MA, Mucha PJ (2009). Mutually-antagonistic interactions in baseball networks.. Physica A.

[15] Petersen AM, Jung WS, Yang JS, Stanley HE (2011). Quantitative and empirical demonstration of the matthew effect in a study of career longevity.. Proc Natl Acad Sci USA.

[16] Ben-Naim E, Redner S, Vazquez F (2007). Scaling in tournaments.. EPL.

[17] Skinner B (2010). The price of anarchy in basketball.. Journal of Quantitative Analysis in Sports.

[18] Albert R, Jeong H, Barabási AL (2000). Error and attack tolerance in complex networks.. Nature.

[19] Pastor-Satorras R, Vespignani A (2001). Epidemic spreading in scale-free networks.. Phys Rev Lett.

[20] Arenas A, Díaz-Guilera A, Kurths J, Moreno Y, Zhou C (2008). Synchronization in complex networks.. Phys Rep.

[21] Szabò G, Fáth G (2007). Evolutionary games on graphs.. Phys Rep.

[22] Perc M, Szolnoki A (2010). Coevolutionary games - a mini review.. BioSystems.

[23] Brin S, Page L (1998). The anatomy of a large-scale hypertextual web search engine.. Comput Netw ISDN Syst.

[24] de Solla Price DJ (1965). Networks of Scientific Papers.. Science.

[25] Chen P, Xie H, Maslov S, Redner S (2007). Finding scientific gems with Google's PageRank algorithm.. Journal of Informetrics.

[26] Bergstrom CT, West J (2008). Assessing citations with the Eigenfactor™ Metrics.. Neurology.

[27] West J, Bergstrom T, Bergstrom CT (2010). Big Macs and Eigenfactor scores: Don't let correlation coefficients fool you.. J Am Soc Inf Sci.

[28] Radicchi F, Fortunato S, Markines B, Vespignani A (2009). Diffusion of scientific credits and the ranking of scientists.. Phys Rev E.

[29] Barrat A, Barthelemy M, Pastor-Satorras R, Vespignani A (2004). The architecture of complex weighted networks.. Proc Natl Acad Sci USA.

[30] Albert R, Barabási AL (2002). Statistical mechanics of complex networks.. Rev Mod Phys.

[31] Newman MEJ (2003). The structure and function of complex networks.. SIAM Review.

[32] Radicchi F, Fortunato S, Castellano C (2008). Universality of citation distributions: Toward an objective measure of scientific impact.. Proc Natl Acad Sci USA.

