# Supplementary material for: Who Is the Best Player Ever? A Complex Network Analysis of the History of Professional Tennis
Source: PLoS One. 2011 Feb 9;6(2):e17249. doi: 10.1371/journal.pone.0017249 (PMC3037277; doi:10.1371/journal.pone.0017249)
Supplement: Table S1 — Top 30 players of the period 1971–1980. (PDF) [file pone.0017249.s001.pdf]

Rank	Player	Country	Hand	Start	End
1	Jimmy Connors	United States	L	1970	1996
2	Bjorn Borg	Sweden	R	1971	1993
3	Ilie Nastase	Romania	R	1968	1985
4	Guillermo Vilas	Argentina	L	1969	1992
5	Arthur Ashe	United States	R	1968	1979
6	Brian Gottfried	United States	R	1970	1984
7	Manuel Orantes	Spain	L	1968	1984
8	Eddie Dibbs	United States	R	1971	1984
9	Harold Solomon	United States	R	1971	1991
10	Stan Smith	United States	R	1968	1985
11	Roscoe Tanner	United States	L	1969	1985
12	Raul Ramirez	Mexico	R	1970	1983
13	Tom Okker	Netherlands	R	1968	1981
14	John Alexander	Australia	R	1968	1985
15	Vitas Gerulaitis	United States	R	1971	1986
16	Ken Rosewall	Australia	R	1968	1980
17	John Newcombe	Australia	R	1968	1981
18	Wojtek Fibak	Poland	R	1974	1988
19	Dick Stockton	United States	R	1968	1984
20	John McEnroe	United States	L	1976	1994
21	Adriano Panatta	Italy	R	1968	1983
22	Jan Kodes	Czech Republic	R	1968	1983
23	Jaime Fillol Sr.	Chile	R	1968	1983
24	Robert Lutz	United States	R	1968	1985
25	Marty Riessen	United States	R	1968	1981
26	Rod Laver	Australia	L	1968	1979
27	Tom Gorman	United States	R	1968	1980
28	Vijay Amritraj	India	R	1970	1993
29	Mark Cox	Great Britain	L	1968	1981
30	Onny Parun	New Zealand	R	1968	1982
