# Supplementary material for: Who Is the Best Player Ever? A Complex Network Analysis of the History of Professional Tennis
Source: PLoS One. 2011 Feb 9;6(2):e17249. doi: 10.1371/journal.pone.0017249 (PMC3037277; doi:10.1371/journal.pone.0017249)
Supplement: Table S2 — Top 30 players of the period 1981–190. (PDF) [file pone.0017249.s002.pdf]

Rank	Player	Country	Hand	Start	End
1	Ivan Lendl	United States	R	1978	1994
2	John McEnroe	United States	L	1976	1994
3	Mats Wilander	Sweden	R	1980	1996
4	Stefan Edberg	Sweden	R	1982	1996
5	Jimmy Connors	United States	L	1970	1996
6	Boris Becker	Germany	R	1983	1999
7	Andres Gomez	Ecuador	L	1979	1993
8	Yannick Noah	France	R	1977	1996
9	Brad Gilbert	United States	R	1981	1995
10	Tomas Smid	Czech Republic	R	1976	1989
11	Henri Leconte	France	L	1980	1996
12	Tim Mayotte	United States	R	1979	1992
13	Anders Jarryd	Sweden	R	1980	1996
14	Miloslav Mecir Sr.	Slovakia	R	1983	1990
15	Kevin Curren	United States	R	1978	1993
16	Aaron Krickstein	United States	R	1983	1996
17	Guillermo Vilas	Argentina	L	1969	1992
18	Joakim Nystrom	Sweden	R	1980	1989
19	Emilio Sanchez	Spain	R	1984	1997
20	Johan Kriek	United States	R	1977	1992
21	Martin Jaite	Argentina	R	1982	1993
22	Jakob Hlasek	Switzerland	R	1982	1996
23	Jimmy Arias	United States	R	1980	1998
24	Pat Cash	Australia	R	1981	1997
25	Ramesh Krishnan	India	R	1973	1993
26	Jose-Luis Clerc	Argentina	R	1977	1989
27	Eliot Teltscher	United States	R	1976	1990
28	Thierry Tulasne	France	R	1979	1991
29	Scott Davis	United States	R	1979	1994
30	Vitas Gerulaitis	United States	R	1971	1986
