# Supplementary material for: Who Is the Best Player Ever? A Complex Network Analysis of the History of Professional Tennis
Source: PLoS One. 2011 Feb 9;6(2):e17249. doi: 10.1371/journal.pone.0017249 (PMC3037277; doi:10.1371/journal.pone.0017249)
Supplement: Table S3 — Top 30 players of the period 1991–2000. (PDF) [file pone.0017249.s003.pdf]

Rank	Player	Country	Hand	Start	End
1	Pete Sampras	United States	R	1988	2002
2	Andre Agassi	United States	R	1986	2006
3	Michael Chang	United States	R	1987	2003
4	Goran Ivanisevic	Croatia	L	1988	2004
5	Yevgeny Kafelnikov	Russian Federation	R	1992	2003
6	Jim Courier	United States	R	1987	2000
7	Richard Krajicek	Netherlands	R	1991	2003
8	Thomas Muster	Austria	L	1984	1999
9	Wayne Ferreira	South Africa	R	1990	2004
10	Thomas Enqvist	Sweden	R	1989	2005
11	Boris Becker	Germany	R	1983	1999
12	Stefan Edberg	Sweden	R	1982	1996
13	Sergi Bruguera	Spain	R	1988	2002
14	Marc Rosset	Switzerland	R	1988	2004
15	Petr Korda	Czech Republic	L	1985	1999
16	Todd Martin	United States	R	1990	2004
17	Cedric Pioline	France	R	1989	2002
18	Michael Stich	Germany	R	1989	1997
19	Alex Corretja	Spain	R	1992	2005
20	Patrick Rafter	Australia	R	1991	2001
21	Magnus Gustafsson	Sweden	R	1986	2001
22	Andrei Medvedev	Ukraine	R	1990	2001
23	Francisco Clavet	Spain	L	1988	2003
24	Marcelo Rios	Chile	L	1994	2003
25	Greg Rusedski	Great Britain	L	1992	2006
26	Fabrice Santoro	France	R	1989	2010
27	Magnus Larsson	Sweden	R	1988	2003
28	Tim Henman	Great Britain	R	1994	2007
29	Alberto Berasategui	Spain	R	1992	2001
30	Albert Costa	Spain	R	1993	2006
