# Supplementary material for: Who Is the Best Player Ever? A Complex Network Analysis of the History of Professional Tennis
Source: PLoS One. 2011 Feb 9;6(2):e17249. doi: 10.1371/journal.pone.0017249 (PMC3037277; doi:10.1371/journal.pone.0017249)
Supplement: Table S4 — Top 30 players of the period 2001–2010. (PDF) [file pone.0017249.s004.pdf]

Rank	Player	Country	Hand	Start	End
1	Roger Federer	Switzerland	R	1998	2010
2	Rafael Nadal	Spain	L	2002	2010
3	Andy Roddick	United States	R	2000	2010
4	Lleyton Hewitt	Australia	R	1997	2010
5	Nikolay Davydenko	Russian Federation	R	2000	2010
6	Ivan Ljubicic	Croatia	R	1978	2010
7	Juan Carlos Ferrero	Spain	R	1978	2010
8	Novak Djokovic	Serbia	R	2004	2010
9	David Nalbandian	Argentina	R	2000	2010
10	Tommy Robredo	Spain	R	1999	2010
11	David Ferrer	Spain	R	2002	2010
12	Fernando Gonzalez	Chile	R	1999	2010
13	Andy Murray	Great Britain	R	2005	2010
14	Carlos Moya	Spain	R	1995	2010
15	Mikhail Youzhny	Russian Federation	R	1999	2010
16	James Blake	United States	R	1998	2010
17	Tommy Haas	United States	R	1996	2010
18	Fernando Verdasco	Spain	L	2002	2010
19	Marat Safin	Russian Federation	R	1997	2009
20	Tomas Berdych	Czech Republic	R	2003	2010
21	Juan Ignacio Chela	Argentina	R	1999	2010
22	Radek Stepanek	Czech Republic	R	1998	2010
23	Andre Agassi	United States	R	1986	2006
24	Robin Soderling	Sweden	R	2001	2010
25	Rainer Schuettler	Germany	R	1995	2010
26	Feliciano Lopez	Spain	L	1998	2010
27	Tim Henman	Great Britain	R	1994	2007
28	Jarkko Nieminen	Finland	L	2000	2010
29	Mardy Fish	United States	R	2000	2010
30	Gaston Gaudio	Argentina	R	1999	2010
