# Supplementary material for: Who Is the Best Player Ever? A Complex Network Analysis of the History of Professional Tennis
Source: PLoS One. 2011 Feb 9;6(2):e17249. doi: 10.1371/journal.pone.0017249 (PMC3037277; doi:10.1371/journal.pone.0017249)
Supplement: Table S5 — Top 30 players of the history of tennis in tournaments played on clay. (PDF) [file pone.0017249.s005.pdf]

Rank	Player	Country	Hand	Start	End
1	Guillermo Vilas	Argentina	L	1969	1992
2	Manuel Orantes	Spain	L	1968	1984
3	Thomas Muster	Austria	L	1984	1999
4	Ivan Lendl	United States	R	1978	1994
5	Carlos Moya	Spain	R	1995	2010
6	Eddie Dibbs	United States	R	1971	1984
7	Jose Higueras	Spain	R	1968	1986
8	Bjorn Borg	Sweden	R	1971	1993
9	Ilie Nastase	Romania	R	1968	1985
10	Andres Gomez	Ecuador	L	1979	1993
11	Alex Corretja	Spain	R	1992	2005
12	Rafael Nadal	Spain	L	2002	2010
13	Jose-Luis Clerc	Argentina	R	1977	1989
14	Sergi Bruguera	Spain	R	1988	2002
15	Mats Wilander	Sweden	R	1980	1996
16	Albert Costa	Spain	R	1993	2006
17	Gaston Gaudio	Argentina	R	1999	2010
18	Juan Carlos Ferrero	Spain	R	1978	2010
19	Harold Solomon	United States	R	1971	1991
20	Emilio Sanchez	Spain	R	1984	1997
21	Adriano Panatta	Italy	R	1968	1983
22	Felix Mantilla	Spain	R	1994	2007
23	Francisco Clavet	Spain	L	1988	2003
24	Balazs Taroczy	Hungary	R	1972	1986
25	Zeljko Franulovic	Croatia	R	1968	1983
26	Tomas Smid	Czech Republic	R	1976	1989
27	Jimmy Connors	United States	L	1970	1996
28	Raul Ramirez	Mexico	R	1970	1983
29	Alberto Berasategui	Spain	R	1992	2001
30	Victor Pecci Sr.	Paraguay	R	1974	1987
