# Supplementary material for: Who Is the Best Player Ever? A Complex Network Analysis of the History of Professional Tennis
Source: PLoS One. 2011 Feb 9;6(2):e17249. doi: 10.1371/journal.pone.0017249 (PMC3037277; doi:10.1371/journal.pone.0017249)
Supplement: Table S6 — Top 30 players of the history of tennis in tournaments played on grass. (PDF) [file pone.0017249.s006.pdf]

Rank	Player	Country	Hand	Start	End
1	Jimmy Connors	United States	L	1970	1996
2	Boris Becker	Germany	R	1983	1999
3	Roger Federer	Switzerland	R	1998	2010
4	John Newcombe	Australia	R	1968	1981
5	John McEnroe	United States	L	1976	1994
6	Pete Sampras	United States	R	1988	2002
7	Tony Roche	Australia	L	1968	1980
8	Stefan Edberg	Sweden	R	1982	1996
9	Roscoe Tanner	United States	L	1969	1985
10	Lleyton Hewitt	Australia	R	1997	2010
11	Ken Rosewall	Australia	R	1968	1980
12	Arthur Ashe	United States	R	1968	1979
13	Stan Smith	United States	R	1968	1985
14	Phil Dent	Australia	R	1968	1983
15	Bjorn Borg	Sweden	R	1971	1993
16	Goran Ivanisevic	Croatia	L	1988	2004
17	Pat Cash	Australia	R	1981	1997
18	Andy Roddick	United States	R	2000	2010
19	Ivan Lendl	United States	R	1978	1994
20	Tim Henman	Great Britain	R	1994	2007
21	Rod Laver	Australia	L	1968	1979
22	Mark Edmondson	Australia	R	1972	1987
23	John Alexander	Australia	R	1968	1985
24	Hank Pfister	United States	R	1975	1988
25	Wally Masur	Australia	R	1980	1995
26	Tim Mayotte	United States	R	1979	1992
27	Vijay Amritraj	India	R	1970	1993
28	Kevin Curren	United States	R	1978	1993
29	Tom Okker	Netherlands	R	1968	1981
30	Greg Rusedski	Great Britain	L	1992	2006
