# Supplementary material for: Who Is the Best Player Ever? A Complex Network Analysis of the History of Professional Tennis
Source: PLoS One. 2011 Feb 9;6(2):e17249. doi: 10.1371/journal.pone.0017249 (PMC3037277; doi:10.1371/journal.pone.0017249)
Supplement: Table S7 — Top 30 players of the history of tennis in tournaments played on hard surfaces. (PDF) [file pone.0017249.s007.pdf]

Rank	Player	Country	Hand	Start	End
1	Andre Agassi	United States	R	1986	2006
2	Jimmy Connors	United States	L	1970	1996
3	Ivan Lendl	United States	R	1978	1994
4	Pete Sampras	United States	R	1988	2002
5	Roger Federer	Switzerland	R	1998	2010
6	Stefan Edberg	Sweden	R	1982	1996
7	Michael Chang	United States	R	1987	2003
8	John McEnroe	United States	L	1976	1994
9	Andy Roddick	United States	R	2000	2010
10	Lleyton Hewitt	Australia	R	1997	2010
11	Brad Gilbert	United States	R	1981	1995
12	Jim Courier	United States	R	1987	2000
13	Brian Gottfried	United States	R	1970	1984
14	Thomas Enqvist	Sweden	R	1989	2005
15	Stan Smith	United States	R	1968	1985
16	Boris Becker	Germany	R	1983	1999
17	Wayne Ferreira	South Africa	R	1990	2004
18	Ilie Nastase	Romania	R	1968	1985
19	Roscoe Tanner	United States	L	1969	1985
20	Tommy Haas	United States	R	1996	2010
21	Rafael Nadal	Spain	L	2002	2010
22	Tim Henman	Great Britain	R	1994	2007
23	Mats Wilander	Sweden	R	1980	1996
24	Yevgeny Kafelnikov	Russian Federation	R	1992	2003
25	Andy Murray	Great Britain	R	2005	2010
26	Fabrice Santoro	France	R	1989	2010
27	Harold Solomon	United States	R	1971	1991
28	Ivan Ljubicic	Croatia	R	1978	2010
29	Marat Safin	Russian Federation	R	1997	2009
30	Aaron Krickstein	United States	R	1983	1996
